# Accuracy of neuropsychological tests and the Neuropsychiatric Inventory in differential diagnosis between Frontotemporal dementia and Alzheimer’s disease

**DOI:** 10.1590/S1980-57642009DN30400012

**Published:** 2009

**Authors:** Valéria Santoro Bahia, Rene Viana

**Affiliations:** 1 MD, PhD, Behavioral and Cognitive Neurology Unit, Department of Neurology, Hospital das Clínicas, University of São Paulo School of Medicine, São Paulo SP, Brazil.; 2 Neuropsychologist, Behavioral and Cognitive Neurology Unit, Department of Neurology, Hospital das Clínicas, University of São Paulo School of Medicine, São Paulo SP, Brazil.

**Keywords:** frontotemporal dementia, Alzheimer’s disease, neuropsychiatric disturbances, differential diagnosis

## Abstract

The differential diagnosis between frontotemporal dementia (FTD) and Alzheimer’s disease (AD) is sometimes difficult.

**Objectives:**

To verify the accuracy of neuropsychological tests and a behavioral disorders scale in the differential diagnosis between FTLD and AD.

**Methods:**

Retrospective data on 12 FTD patients and 12 probable AD patients were analyzed. The scores on neuropsychological tests (MMSE score, reverse digit span, delayed recall for drawings, semantic fluency of animals) and the Neuropsychiatric Inventory (NPI) in both groups were compared.

**Results:**

Both groups had similar performance on neuropsychological tests. All FTD patients and 50% of AD patients had neuropsychiatric abnormalities. The NPI score was 58.0±19.3for the FTD patients, and 3.6±4.7for the AD patients (p< 0.01). Using a NPI cut-off score of 13, the sensitivity and specificity were 100% in this sample. The four most common neuropsychiatric disturbances in FTD patients were: apathy, aberrant motor behavior, disinhibition and eating abnormalities. Apathy and dysphoria/depression were the most common behavioral symptoms among the AD patients.

**Conclusions:**

In this study, NPI was found to be a useful tool for the differential diagnosis between FTD and AD. The neuropsychological tests commonly used in the medical office were unable to distinguish between the two groups.

## Abstract (pt)

O diagnóstico diferencial entre demência frontotemporal (DFT) e doença de Alzheimer (DA) pode ser difícil em alguns casos.

**Objetivos:**

Verificar a acurácia de testes neuropsicológicos e de escala de transtornos comportamentais no diagnóstico diferencial entre DFT e DA.

**Métodos:**

Foram analisados dados retrospectivos de 12 pacientes com o diagnóstico de DFT e de 12 pacientes com DA provável. Os escores dos testes neuropsicológicos (MEEM, extensão de dígitos em ordem inversa, memória tardia de figuras e fluência semântica de animais) e do Inventário Neuropsiquiátrico (INP) dos dois grupos foram comparados.

**Resultados:**

Os dois grupos apresentaram pontuação similar nos testes neuropsicológicos. Todos os pacientes com DFT e 50% dos pacientes com DA apresentavam alterações neuropsiquiátricas. O escore do INP foi 58,0±19,3 em pacientes com DFT e 3,6±4,7 em pacientes com DA provável (p< 0.01). Com o escore de corte de 13 no NPI, a sensibilidade e a especificidade foram de 100%, nesta amostra. Os quatro sintomas neuropsiquiátricos mais comuns em pacientes com DFT foram: apatia, comportamento motor aberrante, desinibição e alteração do comportamento alimentar. Apatia e depressão foram os sintomas comportamentais mais frequentes em pacientes com DA.

**Conclusões:**

Neste estudo, mostramos que o INP é um instrumento útil no diagnóstico diferencial entre DFT e DA enquanto que testes neuropsicológicos geralmente utilizados em consultório não distinguiram os dois grupos.

Frontotemporal Lobar Degeneration (FTLD) is considered the second most prevalent form of neurodegenerative dementia after Alzheimer’s disease (AD) in individuals with early onset dementia.^1-4^

FTLD is rarely diagnosed even by centers specialized in cognitive disorders. For example, the disorder was diagnosed in 5.1% of outpatients at the Behavioral and Cognitive Neurology Unit of Hospital das Clínicas between 1991 and 2001,^5^ and in 5% of patients with presenile dementia in the Cognitive Clinic of Santa Marcelina Hospital.^6^

FTLD includes a spectrum of behavioral and cognitive disorders associated with degeneration of the frontal and anterior temporal lobes. The Consensus Criteria for FTLD distinguished three subtypes of FTLD: the frontotemporal dementia (FTD) that is the most common clinical presentation of FTLD,^2^ semantic dementia (SD), and progressive non-fluent aphasia (PNFA).

FTD is characterized by executive dysfunction and changes in behavior and personality. The core diagnostic features include insidious onset and gradual progression of loss of insight, early decline in social interpersonal behavior, control of personal conduct and early emotional blunting.

AD is the most common cause of neurodegenerative dementia^4,8,9^ and is characterized by progressive impairment of episodic memory and other cognitive domains, such as language, visuospatial perception, praxis or executive functions, generally with well-preserved social skills.^9,10^ Cognitive deficits are primarily responsible for a progressive decline in activities of daily living.^11,12^

Unlike FTD patients, AD patients tend to maintain appropriate social behavior in initial stages of the disease, despite presenting memory deficit. With progression, behavior symptoms arise secondary to cognitive deficits.

FTD is considered a rare disorder indistinguishable from AD or psychiatric disorders in the early clinical stages,^13^ and is frequently underdiagnosed even in specialist settings.^14,15^ Accurate diagnosis of FTD is critical, as it has implications for heritability, prognosis, therapeutics and environmental management of patients.

The objective of the present study was to investigate the accuracy of neuropsychological tests commonly used in the medical office and a behavioral disorders scale in differential diagnosis between FTD and AD.

## Methods

All patients were identified through the Behavioral and Cognitive Neurology Unit of Hospital das Clínicas, in São Paulo, Brazil. Retrospective data on 12 FTD patients that met consensus criteria for FTD, and 12 patients with probable AD according to the criteria developed by the National Institute of Neurological and Communicative Disorders and Stroke and the Alzheimer’s Disease and Related Disorders Association (NINCIDS/ADRDA),^16^ were analyzed.

The diagnoses were based on anamnesis, neurological examination, and neuropsychological assessment that included the Brief Cognitive Battery^17,18^ and, in some cases, the Mattis Dementia Rating Scale.^19^ Activities of daily living were evaluated using the Functional Activities Questionnaire (FAQ).^20^

Patients with other neurological or psychiatry disorders, systemic decompensated disease, motor limitations and hearing and/ or vision impairment were excluded from this study.

All patients underwent structural neuroimaging (CT or MRI) and functional SPECT imaging along with a battery of routine screening blood tests.

The age at onset was defined as the age at which the first symptom consistent with the diagnosis appeared, as reported by the principal informant, while the duration of illness was defined as the interval between age at onset and age on first assessment. Education was considered the number of years of formal education.

The Neuropsychiatric Inventory (NPI)^21,22^ was used to assess behavioral disorders. The NPI is based on a structured interview with a caregiver of the patient. We considered caregivers those who were in daily contact with the patient and who oversaw their treatment.

The NPI assesses 12 types of neuropsychiatric disturbances (Delusions, Hallucinations, Agitation/Aggression, Dysphoria/depression, Anxiety, Euphoria, Apathy, Disinhibition, Irritability/lability, Aberrant motor activity, Night-time behavior disturbances and Appetite/eating abnormalities). The Caregiver rates the frequency and severity of each abnormality as well as distress associated with each behavioral symptom. The total score for each domain is calculated by multiplying the frequency by the severity, and the total score is the sum of the scores of all domains.

To evaluate the cognitive performance of these patients, we chose the following neuropsychological tests: MMSE score (dementia screening), reverse digit span, semantic fluency of animals (executive function), delayed recall for drawings (memory).

Descriptive statistics analyses were performed using BioEstat 3.0 software. Comparisons of frequency data for the FTD and AD groups were done using the Mann Whitney test. Statistical significance was set at p< 0.05.

## Results

Complete demographic features of the patients are shown in Table 1. The groups were matched for gender, duration of disease and schooling.

**Table 1:** Demographic characteristics of the sample.

Demographic features	FTD	AD	p
N	12	12	
Gender	7W:5M	8W:4M	0.80
Age (years)	55.9±7.5	74.8±7.3	< 0.001
Age at onset (years)	50.5±9.3	72.4±7.1	< 0.001
Schooling (years)	8.2±5.3	5.7±4.7	0.08
Duration of disease (years)	3.6 ±1.8	2.6±1.4	0.21

N, number of patients; W, Women; M, Men; FTD, Frontotemporal Dementia; AD, Alzheimer's disease.

Regarding neuropsychological assessment, both groups had similar performance in terms of MMSE score, reverse digit span, delayed recall for drawings, semantic fluency of animals (Table 2).

**Table 2:** Neuropsychological tests and NPI.

Tests	FTD	AD	p
MMSE	13.3±8.9	16.5±5.2	0.26
Reverse Digit span	1.6±1.5	1.63±1.2	0.94
Delayed recall of drawings	3.9±3.4	1.2±1.4	0.09
Semantic fluency	5.0±3.8	7.8±3.3	0.09
NPI	58.0±19.3	3.6±4.7	<0.001

MMSE, Mini Mental State Examination; NPI, Neuropsychiatric Inventory; FTD, Frontotemporal Dementia; AD, Alzheimer's disease.

With respect to NPI, all FTD patients and 50% of AD patients had neuropsychiatric abnormalities. The score was 58.0±19.3for the FTD patients, and 3.6±4.7for the AD patients (p< 0.01). Using a NPI cut-off score of 13, the sensitivity and specificity were 100%.

**Figure 1:**
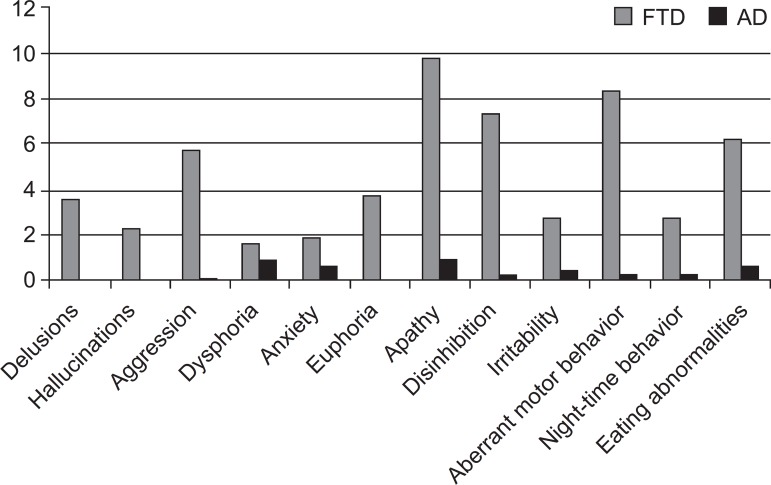
Neuropsychiatric disturbances in FTD and AD patients.

Comparisons of the behaviors investigated on the NPI in both groups are shown in Figure 1. The four most common neuropsychiatric disturbances in FTD patients were: apathy, aberrant motor behavior, disinhibition and eating abnormalities. Apathy and dysphoria/depression were the most common behavioral symptoms among the AD patients.

No statistically significant difference was found between the two groups with respect to dysphoria/depression, anxiety, hallucinations, irritability and night-time behavior.

## Discussion

In this study, the NPI efficiently distinguished FTD from AD patients revealing marked behavioral differences between the two groups. The sensitivity and specificity were 100% for a cut-off score of 13. Apathy, aberrant motor behavior, disinhibition and eating abnormalities occurred very frequently in FTD patients whereas apathy and dysphoria/depression were the most common behavioral symptoms among the AD patients. Only 50% of AD patients had behavioral abnormalities.

Neuropsychological tests (MMSE score, reverse digit span, delayed recall for drawings, semantic fluency of animals) were not effective in this differential diagnosis.

In general, the earliest manifestations of FTD are behavioral changes and executive dysfunctions, while for AD there is a predominance of impaired episodic memory and visuospatial skills.^23^ Some FTLD patients have memory complaints, usually related to the dysexecutive syndrome or to word finding difficulties arising from language dysfunction,^24,25^ although there is some evidence of hippocampal atrophy in SD and FTD patients.^26-28^

In 2007, Hutchinson and Mathias^29^ published a meta-analysis which included 94 studies and showed that scores of orientation, memory, visuomotor function, language and global cognitive ability were useful in achieving a differential diagnosis between FTD and AD, and that a large overlap can occur in the test performance of patients. Thus, these authors recommended that cognitive tests be used cautiously and in conjunction with anamnesis, behavioral scales, neuroimaging, and information from caregivers in reaching a differential diagnosis.

Thompson et al..^30^ 2005, emphasized that numerical scores on neuropsychological tests alone are of limited value in differentiating FTD and AD, but performance characteristics and error types enhance the distinction between the two disorders. Hence for this differential diagnosis, qualitative data are more important than quantitative data.

In AD patients, mood symptoms (i.e. depression, anxiety and apathy) generally develop early in the course of the disease, while psychotic symptoms and agitation characterize the middle to late stages of dementia.^31^

Behavioral assessment is very important for reaching a diagnosis of FTD in its early stages. Liscic et al.^23^ evaluated 48 FTLD patients and 27 AD patients with confirmation by autopsy. They showed that the presence of impulsivity, disinhibition, social withdrawal and progressive nonfluent aphasia distinguished individuals with FTLD from those with AD. The performance measured on tests of executive dysfunction was similar for the FTLD and AD groups.

Several instruments are available for assessing behavioral symptoms in patients with dementia including the Frontal Behavioral Inventory (FBI), which was devised specifically to assess the behavioral disturbance in FTLD.^32-34^ Kertesz et al.,^35^ 2000, administered the FBI in patients with FTD, PNFA, AD, vascular dementia and depressive disorder. They demonstrated that the scale correctly classified 92.7% of the FTD patients and demonstrated high internal consistency and inter-rater reliability.

The NPI^21,22^ is the most frequently used questionnaire for scoring behavioral and psychotic symptoms in dementia as well as the distress experienced by caregivers for each neuropsychiatry symptom. Recently, the Brazilian Portuguese version was validated for use in Brazil in AD patients.^36^ The cited study showed a high frequency of apathy among AD patients (mean score=5.31±4.91) while in our study, the frequency of apathy was much lower in this patient group (mean score=0.9±2.3) even though apathy and dysphoria/depression was found to be the most frequent behavioral symptom. This finding may be explained by the small number of patients evaluated in our study. Caputo et al. evaluated 690 AD patients and showed that apathy was more frequent than depression in AD patients and was more evident in late stages of the disease.^31^

Our study has some methodological limitations. Firstly, this was a retrospective study, and in some cases specific features may not have been reported by the caregiver. Secondly, the study lacked autopsy confirmation and the number of patients was very small.

In sum, our findings support the use of the NPI for the differential diagnosis between AD and FTD and confirm that the neuropsychological tests commonly used in the medical office were unable to distinguish between the two groups.
